# Bioinformatics analysis of the common targets of miR-223-3p, miR-122-5p, and miR-93-5p in polycystic ovarian syndrome

**DOI:** 10.3389/fgene.2023.1097706

**Published:** 2023-02-16

**Authors:** Liping Zou, Qiwen Feng, Wei Xia, Changhong Zhu

**Affiliations:** Institute of Reproductive Health, Tongji Medical College, Huazhong University of Science and Technology, Wuhan, China

**Keywords:** PCOS, miRNA, bioinformatics analysis, biomarkers, granulosa cells

## Abstract

Polycystic ovarian syndrome (PCOS) is one of the most common gynecological endocrine disorders. MicroRNAs (miRNAs) play extensive roles in the pathogenesis of PCOS and can serve as potential diagnostic markers. However, most studies focused on the regulatory mechanisms of individual miRNAs, and the combined regulatory effects of multiple miRNAs remain unclear. The aim of this study was to identify the common targets of miR-223-3p, miR-122-5p, and miR-93-5p; and assess the transcript levels of some of these targets in PCOS rat ovaries. Transcriptome profiles of granulosa cells from PCOS patients were obtained from the Gene Expression Omnibus (GEO) database to identify differentially expressed genes (DEGs). A total of 1,144 DEGs were screened, 204 of which were upregulated and 940 were downregulated. According to the miRWalk algorithm, 4,284 genes were targeted by all three miRNAs at the same time, and intersection with DEGs was used to obtain candidate target genes. A total of 265 candidate target genes were screened, and the detected target genes were subjected to Gene ontology (GO) and KEGG pathway enrichment, followed by PPI network analysis. Then, qRT-PCR was used to determine the levels of 12 genes in PCOS rat ovaries. The expressions of 10 of these genes were found to be consistent with our bioinformatics results. In conclusion, JMJD1C, PLCG2, SMAD3, FOSL2, TGFB1, TRIB1, GAS7, TRIM25, NFYA, and CALCRL may participate in the development of PCOS. Our findings contribute to the identification of biomarkers that may promote the effective prevention and treatment of PCOS in the future.

## Introduction

PCOS is a common endocrine and metabolic disease in women of reproductive age. According to the Rotterdam diagnostic criteria, it encompasses all combinations of ovulation dysfunction, hyperandrogenemia, and polycystic ovary morphology (Ehrmann, 2005; Rosenfield and Ehrmann, 2016). Patients with PCOS often have a range of other health problems, including infertility, insulin resistance (IR), and obesity (Patel, 2018). Several studies suggest that genetic, environmental, and epigenetic factors may play important roles in the pathogenesis of PCOS (Escobar-Morreale, 2018), but the exact mechanism remains largely unclear.

MiRNAs are small endogenous single-stranded non-coding RNA molecules consisting of 18–25 nucleotides that regulate gene expression at the post-transcriptional level (Fabian et al., 2010). It has been reported that miRNAs are involved in various signaling pathways in PCOS, including amino acid metabolism, hormone regulation, cell differentiation, etc. (Imbar and Eisenberg, 2014; Mu et al., 2021). Differentially expressed miRNAs play important roles in PCOS pathogenesis and serve as potential diagnostic markers (Deswal and Dang, 2020). In addition, miRNAs regulate many biological processes associated with obesity, including adipogenesis, insulin secretion, and glucose uptake (Butler et al., 2020). Inflammation of adipose tissue in obese patients contributes to obesity-related metabolic dysfunction, such as insulin resistance and type 2 diabetes (Wu and Ballantyne, 2020).

Upregulation of miR-223 and miR-93 was detected in adipose tissue of PCOS patients (Chen et al., 2013; Udesen et al., 2020), while miR-93 was also significantly upregulated in the ovarian cortex and follicular fluid of PCOS patients (Jiang et al., 2015; Butler et al., 2019). Interestingly, miR-223 and miR-93 were not only overexpressed in adipose tissue of PCOS patients, but also in control patients with IR, with which it was positively correlated *in vivo* (Chen et al., 2013; Chuang et al., 2015). MiR-223 and miR-93 downregulate GLUT4 expression and inhibit insulin-stimulated glucose uptake in adipocytes, suggesting that they may play an important role in other IR-related diseases such as T2DM and obesity. Increased serum miR-122 levels were found in PCOS patients with impaired glucose metabolism (Jiang et al., 2016). Elevated miR-122 in circulation was positively associated with obesity and IR in young adults (Wang et al., 2015). Furthermore, miR-122 and miR-223 were found to be increased in obesity or hyperglycemia, and their intracellular roles are related to the development of IR (Murri et al., 2018; Udesen et al., 2020).

Experimental studies confirmed that the expression of miR-223-3p, miR-122-5p and miR-93-5p was significantly upregulated in PCOS (Chen et al., 2013; Jiang et al., 2016). This may indicate that these differential miRNAs are key molecules involved in the pathological process of PCOS. However, most studies focused the regulation mechanism of individual miRNAs, while the combined regulatory effect of multiple miRNAs remained unclear.

Each miRNA may affect hundreds of targets, while itself being regulated by several distinct miRNAs. Since the efficacy of single markers is limited, and multi-marker-based models can provide more reliable information for the diagnosis and therapeutic management of PCOS, we aimed to identify the common targets of miR-223-3p, miR-122-5p, and miR-93-5p; and assess the transcript levels of some of these targets in PCOS rat ovaries.

## Materials and methods

### Microarray data and DEGs screening

The gene expression profile GSE34526 was acquired from the GEO website. GSE34526 contained 3 granulosa cells samples (GSM850527-GSM850529) from female controls and 7 granulosa cells samples (GSM850530-GSM850536) from PCOS patients, based on the GPL570 [HG-U133_Plus_2] Affymetrix Human Genome U133 Plus 2.0 Array. DEGs between PCOS and normal samples were obtained from the GEO database by GEO2R analysis (https://www.ncbi.nlm.nih.gov/geo/geo2r). A *p*-value <0.05 and |logFC| > 2 as used as the DEGs cut-off criteria. A volcano plot was generated to visualize DEG expression changes using GraphPad Prism 8.0 software (GraphPad, United States).

### Prediction of target genes

The miRWalk Version 3.0 (https://mirwalk.umm.uni-heidelberg.de), which is linked to three online databases (TargetScan, miRDB, and miRTarBase), stores predicted data including experimentally verified miRNA-target interactions. The targets genes of miR-223-3p, miR-122-5p, and miR-93-5p were downloaded from the miRWalk 3.0 database, and the intersection genes were selected for further analysis. Then, the overlapping genes among DEGs and targets genes of the miRNAs were obtained using the Venn diagrams tool (https://bioinformatics.psb.ugent.be).

### Functional enrichment analysis of target genes

GO functional enrichment and KEGG pathway analyses were performed for the gene overlaps using Metascape (https://metascape.org). Metascape always uses the latest data, which integrates data sources from GO, KEGG, UniProt, and DrugBank to achieve pathway enrichment and biological process annotation. *p* < 0.01 was considered statistically significant.

### PPI network analysis of target genes

The Search Tool for the Retrieval of Interacting Genes (STRING) online database (https://string-db.org) was used to predict the protein-protein interaction (PPI) network specific to target genes. Then, PPI networks were visualized using Cytoscape version 3.8.2 (https://cytoscape.org) and the clusters (highly interconnected regions) in the PPI network were identified using the Cytoscape plugin MCODE.

### Genes related to obesity with PCOS

The DEGs related to obesity in PCOS were identified in the dataset of GSE80432, including four granulosa cell samples (GSM2127203-GSM2127204, GSM2127215-GSM2127216) from normal weight PCOS patients and four granulosa cell samples (GSM2127209, GSM2127211-GSM2127212, GSM2127214) from obese PCOS patients. The datasets were analyzed on the GPL6244 [HuGene-1_0-st] Affymetrix Human Gene 1.0 ST Array [transcript (gene) version]. The *p*-value <0.05 and Fold Change >1.5 were set as the cut-off criteria for DEGs. The Venn diagram tool was used to identify the genes shared between DEGs and targets genes of miRNAs.

### TFs related to miR-223-3p, miR-122-5p and miR-93-5p

The prediction of TFs related to miR-223-3p, miR-122-5p, and miR-93-5p was conducted using TransmiR v2.0 (https://www.cuilab.cn/transmir), which is a database for TF-miRNA regulatory association. Subsequently, the TFs involved in PCOS shared between DEGs and predicted TFs were screened using the Venn diagram tool.

### Animal model

Female Sprague–Dawley rats (*n* = 20; 21 days old) were obtained from the Hubei Provincial Center for Disease Control and Prevention (Wuhan, China). Animal experiments were approved by the Animal Ethics Committee of Tongji Medical College, Huazhong University of Science and Technology (Wuhan, China). PCOS was induced by injection of DHEA (60 mg/kg body weight) dissolved in 0.2 mL sesame oil. The control group was injected with the vehicle (0.2 mL sesame oil). Daily treatment was continued for up to 21 days. In this study, we used a rat model that exhibits reproductive and metabolic abnormalities similar to human PCOS to uncover molecular mechanisms.

### Quantitative real-time polymerase chain reaction (qRT-PCR)

Total RNA was extracted from the ovaries of PCOS rats using TRIzol reagent (Ambion). The cDNA as generated using the PrimeScript™ RT reagent kit or miRNA First-Strand Synthesis kit (Takara). The qRT-PCR was performed using qPCR SYBR Green Master Mix (Vazyme). GAPDH and U6 were used as internal controls for mRNA and miRNA expression, respectively. The relative expression of the mRNAs and miRNAs was calculated using the 2^−(ΔΔCt)^ method.

### Statistical analysis

Data are presented as the means ± SD. Data were analyzed using Student’s t-test. Differences with *p*-values <0.05 were considered statistically significant. The results were analyzed and visualized using GraphPad Prism 8.0 software (GraphPad Inc, United States).

## Results

### Identification of DEGs and target genes in PCOS

**FIGURE 1:**
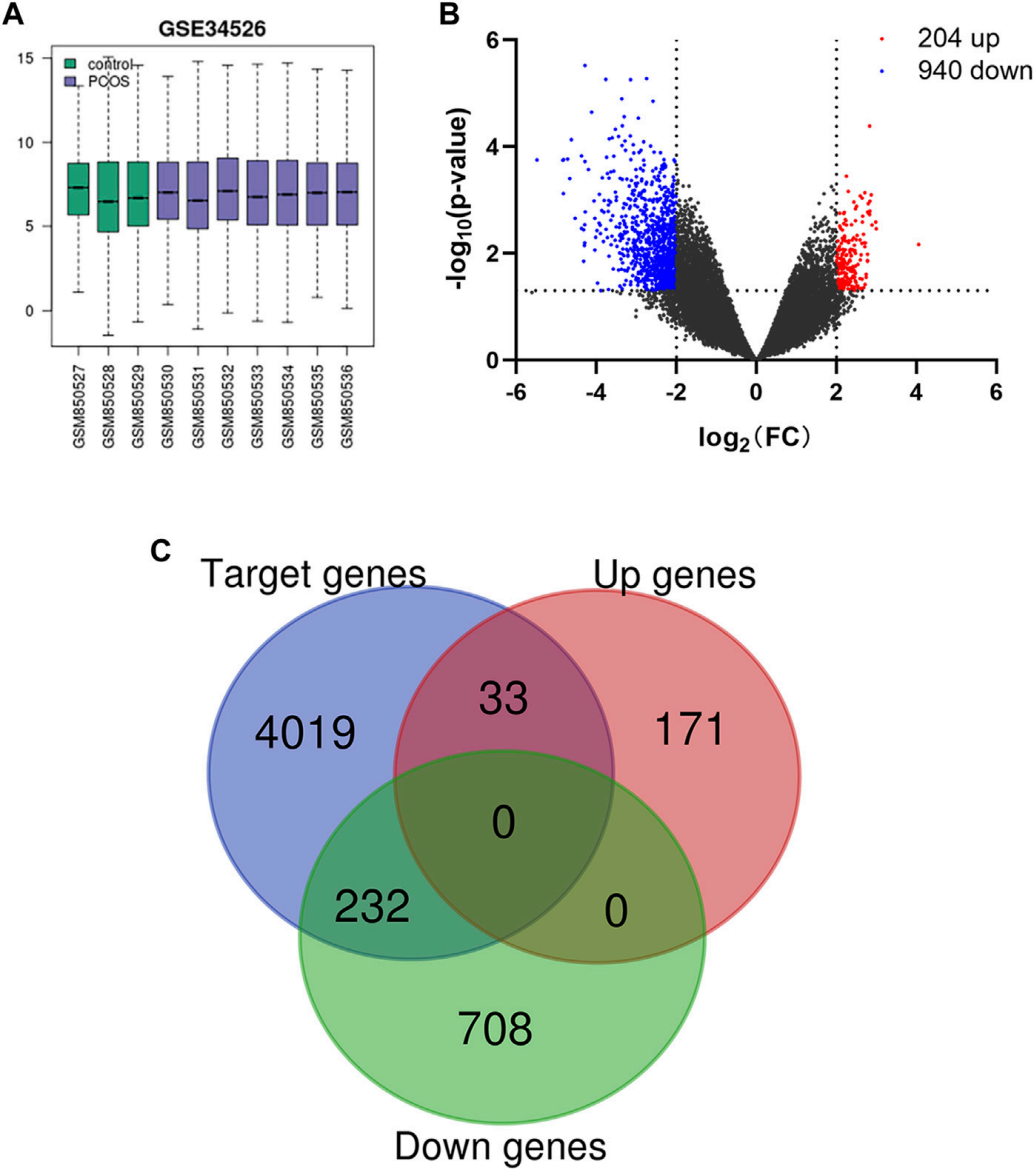
**(A)** Box plot of gene expression profiles after standardization. **(B)** Volcano plot of DEGs in PCOS. Red dots represent upregulation, blue dots represent downregulation, and gray dots represent no differential expression. **(C)** Venn diagram of DEGs and miRNA target genes.

After normalization of the microarray data from GSE34526 (Figure 1A), a total of 1,144 DEGs were identified in PCOS patients compared with female controls, consisting of 204 upregulated genes and 940 downregulated genes (Figure 1B). The downstream target genes associated with miRNAs were identified, including 6,653 for miR-223-3p, 12,132 for miR-122-5p, and 15,170 for miR-93-5p. Among them, 4,284 target genes were shared among all three miRNAs. Finally, a total of 265 overlapping genes shared between DEGs and target genes were obtained, including 33 upregulated genes and 232 downregulated genes (Figure 1C).

### GO and KEGG analysis of candidate target genes in PCOS

**FIGURE 2:**
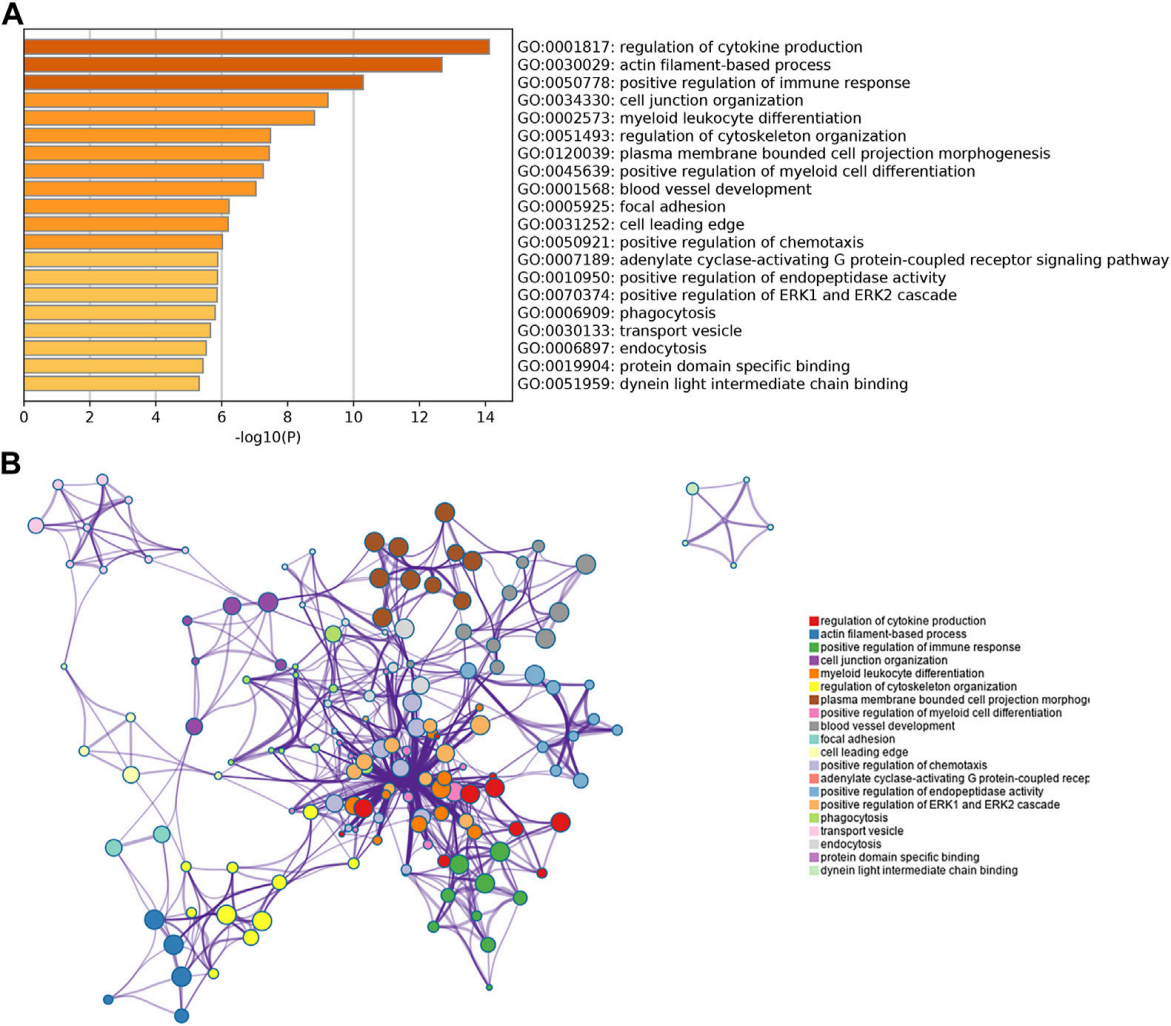
GO enrichment analysis of overlapping genes obtained from the intersection between DEGs and miRNA target genes. **(A)** Bar chart of the most highly enriched terms. **(B)** Network of the most highly enriched terms.

**FIGURE 3:**
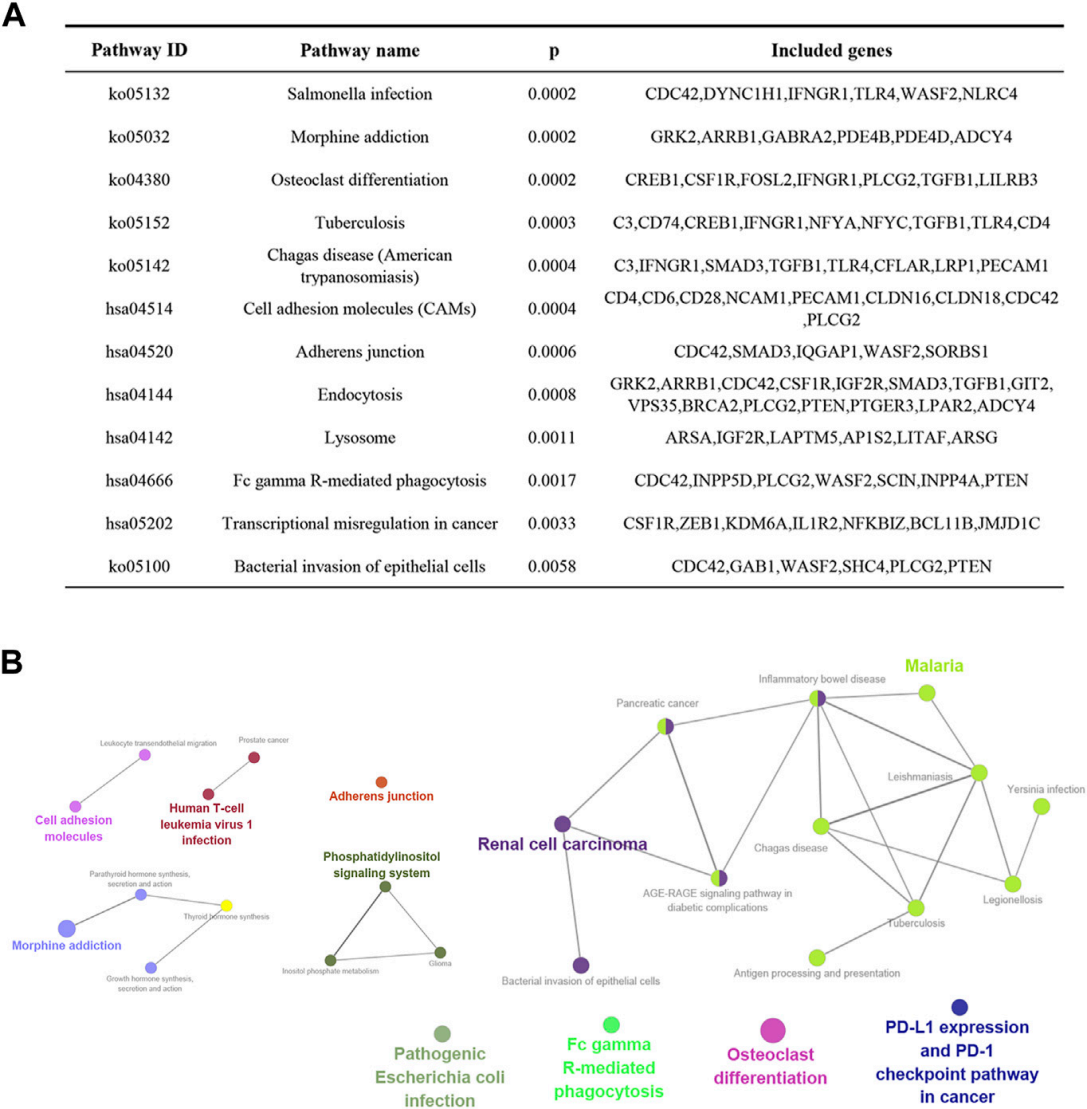
KEGG enrichment analysis of overlapping genes obtained from the intersection between DEGs and miRNA target genes. **(A)** Genes involved in enriched signaling pathways. **(B)** Network of the most highly enriched signaling pathways.

To further explore the function of the identified target genes, GO term and KEGG pathway enrichment analyses were performed. GO analysis was divided into the MF, CC, and BP categories (Figure 2). The results showed that 265 genes were mainly enriched in the regulation of cytokine production, actin filament-based processes, positive regulation of the immune response, cell junction organization, and myeloid leukocyte differentiation. Moreover, 12 KEGG pathways were overrepresented, including *salmonella* infection, morphine addiction, osteoclast differentiation, cell adhesion molecules, adherens junction, and endocytosis (Figure 3).

### PPI network construction and hub gene selection

**FIGURE 4:**
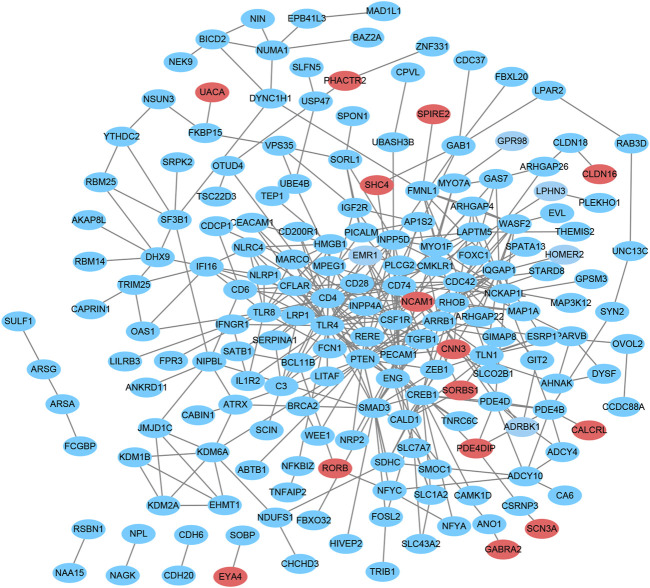
Protein-protein interaction network analysis of the overlapping genes. The PPI network consists of 265 nodes and 389 edges. Red indicates upregulation, and blue indicates downregulation.

To explore the interactions among proteins encoded by the identified target genes, a PPI network was constructed, including 265 nodes and 389 edges (Figure 4). The three downregulated genes JMJD1C, PLCG2, and SERPINA1 were selected as hub genes based on enrichment degree ≥2 as the cutoff criterion.

### TFs associated with miR-223-3p, miR-122-5p and miR-93-5p

A total of 229 TFs associated with miR-223-3p, miR-122-5p, and miR-93-5p were downloaded from the TransmiR database. Compared with normal granulosa cells, 7 TFs were differentially downregulated in PCOS, including TGFB1, SMAD3, FOSL2, JMJD1C, CREB1, TRIM25, and NFYA.

### DEGs related to obesity in PCOS

To further clarify the potential mechanisms of miRNAs related to adipogenesis in obese patients with PCOS, the genes that were differentially expressed in granulosa cells and associated with obesity were selected for further analysis. Based on the intersection between 265 genes and DEGs from the GSE80432 dataset, one upregulated and four downregulated genes were obtained, including CALCRL, TGFB1, TRIB1, GAS7, and FOSL2. TGFB1 and FOSL2 are both major transcription factors and closely related to the pathological process of obesity in PCOS.

### Validation of the identified DEGs in a rat model of PCOS

**FIGURE 5:**
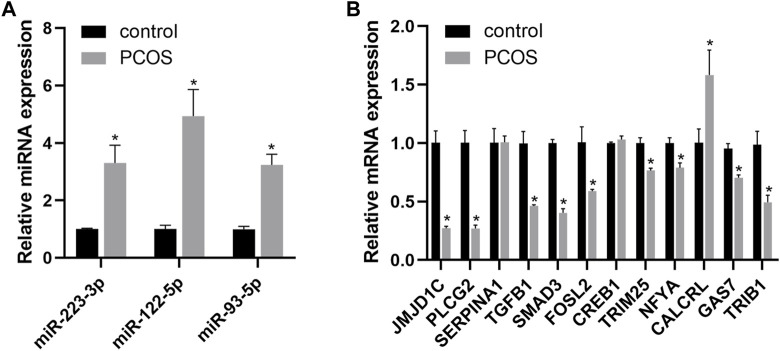
The qPCR verification results of three miRNAs **(A)** and twelve candidate target genes **(B)** in the rat model of PCOS. An asterisk indicates a significant difference between the two groups (∗*p* < 0.05) according to Student’s t-test.

To validate the results of bioinformatics analysis, we detected the expression of three miRNAs and 12 predicted target genes using qRT-PCR in PCOS model rats. As shown in Figure 5, miR-223-3p, miR-122-5p, and miR-93-5p were significantly upregulated in the PCOS group compared with control group. JMJD1C, PLCG2, SMAD3, FOSL2, TGFB1, TRIB1, GAS7, TRIM25, and NFYA were downregulated, while CALCRL was highly expressed in PCOS rats. Furthermore, SERPINA1 and CREB1 were not validated in PCOS rats.

## Discussion

PCOS is a common endocrine and metabolic disorder that can easily lead to ovulation dysfunction in women of childbearing age, resulting in infertility. The development of oocytes is inseparable from the role of follicular granulosa cells, which provide nutrients for oocytes through gap junctions and regulate oocyte development through paracrine signals (Clarke, 2018). In this study, a total of 265 candidate target genes were screened by integrating the information of DEGs and miRNA target genes in granulosa cells of PCOS patients with insulin resistance, among which 7 TF genes and five target genes closely related to obesity were identified. The function of candidate target genes was analyzed and the enriched GO terms included regulation of cytokine production, actin filament-based process, positive regulation of immune response, cell junction organization, and myeloid leukocyte differentiation.

We selected three DEGs as hub genes, the top three of which were JMJD1C, SERPINA1, and PLCG2. It stands to reason that miRNAs may play a major role in PCOS through these key target genes. We verified their transcriptional levels in a rat model of PCOS and found that the expression of JMJD1C and PLCG2 was downregulated, while the expression of SERPINA1 was unchanged. The mechanism may be that miRNA mediates the attenuation of JMJD1C and PLCG2 mRNA, hindering the translation of SERPINA1 mRNA without affecting its stability. Among them, JMJD1C and SERPINA1 play important roles in adipogenesis. JMJD1C regulates the induction of adipogenic transcription factors *via* H3K9me2 (Buerger et al., 2017) and promotes adipogenesis *in vivo* to increase liver and plasma triglyceride levels (Viscarra et al., 2020). SERPINA1, which may serve as an important molecular marker of obesity, affects energy expenditure by regulating the AMPK pathway and promotes the development of obesity-related metabolic complications (Mansuy-Aubert et al., 2013). PLCG2 regulates oxidative stress in hypoxic-ischemic encephalopathy, and activated PLCG2 signaling to attenuate oxidative stress-induced neuronal degeneration and apoptosis (Hu et al., 2020). Ovarian oxidative stress imbalance is a key feature of PCOS, suggesting that downregulation of PLCG2 is closely related to PCOS (Murri et al., 2013).

The selected transcription factors included TGFB1, SMAD3, FOSL2, JMJD1C, CREB1, TRIM25, and NFYA. The mRNA levels of TGFB1, SMAD3, FOSL2, TRIM25, and NFYA were decreased in our animal model. TGFB1 partially inhibits adipogenesis through SMAD3 (Tsurutani et al., 2011), and downregulation of the TGFB1 signaling pathway may promote adipogenesis by altering the expression of adipogenic genes to change the PCOS phenotype (Li and Wu, 2020). Furthermore, androgens were found to induce ovarian fibrosis through the TGFB1 signaling pathway in a rat model of PCOS (Wang et al., 2018). It is speculated that TGFB1 mainly regulates adipogenesis in PCOS and has less effect on ovarian fibrosis. FOSL2 is known to be a key regulator of adipokine LEP expression in obese mice and humans (Wrann et al., 2012). TRIM25 enhances the antioxidant defense by activating Nrf2 (Liu et al., 2020), and downregulation of TRIM25 may contribute to the oxidative stress imbalance phenotype of PCOS. NFYA regulates the gene expression of adiponectin in adipose tissue, one of the adipokines secreted by adipocytes that regulate energy homeostasis related to insulin sensitivity (Park et al., 2004).

Although CALCRL gene expression was reported to be lower in the adipose tissue of obese patients (Aguilera et al., 2015; Kim et al., 2020), CALCRL transcription was increased in the rat PCOS model. This difference may be caused by differences between species. TRIB1 knockout mice exhibit obesity and increased lipid accumulation in their livers (Bauer et al., 2015). TRIB1 can promote adipose tissue thermogenesis by regulating mitochondrial function (Zhang et al., 2021). PCOS patients have lower brown adipose tissue (BAT) activity and reduced BAT thermogenesis, which is associated with increased insulin resistance (Robinson et al., 1992; Shorakae et al., 2019). The expression of the GAS7 gene was found to be decreased in the ovaries of obese women, and its transcript was also downregulated in the ovaries of DHT-treated rats (Ruebel et al., 2017), which was consistent with our prediction. These results demonstrate the accuracy of predicting miRNA target genes and demonstrate that miRNAs regulate the pathological process of PCOS.

Although this study revealed the common targets of three miRNAs in PCOS using bioinformatics analyses and validated them using a rat model, there are still some limitations in the present study. Firstly, the GSE34526 and GSE80432 datasets were collected from granulosa cells samples, which may be the reason why the expression of SERPINA1 and CREB1 in PCOS rats was inconsistent with the bioinformatics analyses. In addition, the miRNA–mRNA relationships in PCOS were based on target prediction. However, the fact that the predicted targets are dysregulated in the PCOS rat model does not mean that they were targeted by the three miRNAs, which needs further experimental validation. Thus, a knockdown or overexpression of the miRNAs in granulosa cells and assessment of the mRNA and protein levels of the predicted targets would have yielded more reliable information.

In conclusion, JMJD1C, PLCG2, SMAD3, FOSL2, TGFB1, TRIB1, GAS7, TRIM25, NFYA, and CALCRL were found to be potential common targets of the three miRNAs. These possible targets were downregulated in PCOS rat ovaries, and so may serve as biomarkers for PCOS diagnosis and treatment. Instead of relying solely on the target gene prediction of a single miRNA, we used multiple miRNAs identified in clinical samples to predict common target genes, which provides strong evidence for the involvement of miRNAs in PCOS and broadens our understanding of gene expression changes in PCOS.

## Data Availability

The original contributions presented in the study are included in the article/Supplementary Material, further inquiries can be directed to the corresponding author.
